# Correlation of affective temperament and psychiatric symptoms in palliative care cancer patients

**DOI:** 10.1007/s00508-018-1400-6

**Published:** 2018-10-22

**Authors:** Matthias Unseld, Benjamin Vyssoki, Ines Bauda, Marlene Felsner, Feroniki Adamidis, Herbert Watzke, Eva Masel, Nestor D. Kapusta

**Affiliations:** 1 Department for Palliative Care and Clinical Oncology, Institute for Internal Medicine I, Comprehensive Cancer Center, Medical University of Vienna, Vienna, Austria; 2 Department for Psychoanalysis and Psychotherapy, Medical University of Vienna, Waehringer Guertel 18–20, 1090 Vienna, Austria

**Keywords:** Tumor patients, Psychiatric disorders, Temperamental trait, Quality of life

## Abstract

**Background:**

Psychiatric symptoms are common in terminally ill patients. Studies linking temperamental traits and psychiatric disorders in patients suffering from advanced disease are rare. This study investigated the influence of temperament on depression and anxiety in palliative care cancer patients.

**Methods:**

A total of 53 patients at the palliative care unit (PCU) of the Medical University of Vienna were enrolled in the study. Patients filled out the TEMPS-M and the brief PHQ-9 questionnaires to examine associations between temperament, psychiatric symptoms and sociodemographic parameters.

**Results:**

Pain (67%), anorexia (58%), constipation (42%) and nausea/vomiting (40%) were the most prevalent self-reported symptoms. Self-reported symptoms of depression were less commonly reported (21.8%) than unveiled by the structured assessment by the PHQ-9 questionnaire: 26.4% (*n* = 14) showed mild symptoms of depression and 64.1% (*n* = 34) had a major depressive disorder (MDD) according to PHQ-9. The depressive and cyclothymic temperaments showed significant associations with depressive (both: *p* < 0.001) as well as symptoms of anxiety (*p* = 0.002; *p* = 0.036). Furthermore, the anxious temperament was significantly associated with symptoms of depression (*p* = 0.027).

**Conclusions:**

Mood disorders are common in palliative care patients, as the majority of the patients were suffering from MDD. The depressive, cyclothymic and anxious temperaments were found to be correlated with depressive and anxious symptoms. A sensitization in this field might bring further improvements for the quality of life of palliative care patients and help to appropriately address psychiatric symptoms in palliative care.

## Introduction

Patients suffering from progressive diseases with limited life-expectancy are in need of multidisciplinary and comprehensive medical care. Treatment efforts are frequently focused on physical symptoms while comorbid psychiatric conditions are often overlooked and therefore untreated in 60–80% of the cases [1–3]. Up to 60% of terminally ill patients experience psychiatric symptoms at least at some point of the disease [3]. For example, studies on cancer patients reported prevalence rates for depression of between 10% and 50% [1, 2]. According to Kerrihard et al. [4] 15–28% of cancer patients suffer from anxiety and general fears, which often occur with somatic symptoms, such as fatigue, pain, vomiting, insomnia and death anxiety [5]. Depressive and anxiety symptoms are 3 times more likely to occur in the last 3 months of life than 1 year before death [5] and require appropriate recognition and treatment. The co-occurrence of depression with cancer is not only associated with reduced quality of life but also with decreased adherence to treatment, increased numbers of inpatient stays and a general poorer survival [5–7]. Correct interpretation of psychiatric symptoms in advanced cancer patients can be challenging as many symptoms, such as fatigue and loss of interest and appetite can be either linked to the primary disease, side effects of chemotherapy or due to a mood disorder.

Individual behavioral and emotional reactivity patterns as characterized through the concept of temperament [8] play an essential role as moderators of major depressive disorder (MDD) [9] and anxiety disorders [10] in psychiatric patient populations. Based on the well-established 5‑factor concept of affective temperaments (i.e. anxious, depressive, hyperthymic, cyclothymic and irritable) [11, 12], the hyperthymic temperament has been shown to be a resilience factor for MDD [13]. It remains open whether and to what extent the temperament has an impact on terminally ill patients, therefore the associations of temperament with depression as well as anxiety were analyzed in a cross-sectional study.

To the best of our knowledge, no studies have exclusively addressed the role of temperament as a risk factor in the development of depression and anxiety among patients admitted to PCUs. The results of this study could provide new options for treatment and enhance the quality of life in palliative care patients. The assessment of temperament and psychiatric symptoms in patients in a PCU could enable the multidisciplinary team to recognize the patients’ needs more appropriately and to allocate the necessary psychosocial support.

## Methods

The study was performed at the palliative care ward of the Division of Palliative Care at the Department of Medicine I at the Medical University of Vienna, Austria.

### Ethical considerations

This study was approved by the institutional Ethics Committee of the Medical University of Vienna and Vienna General Hospital (AKH) under the reference number EC 1472/2015.

### Sample

Data were collected between May and September 2015. During this period a total of 94 inpatients were admitted to the PCU. All were subsequently approached to participate in the study. In total, 55 patients (response rate 58.5%) participated and filled in the questionnaires, which included general sociodemographic questions, the brief TEMPS-M questionnaire (TEMPS: Temperament Evaluation of Memphis, Pisa, Paris and San Diego-autoquestionnaire version) (*n* = 48, response rate 51%) and the Patient Health Questionnaire (PHQ-9, *n* = 53, response rate 56%). The inclusion criteria were age over 18 years, ability and willingness to participate by signing an informed consent declaration. Exclusion criteria were mental impairment or patients who could not sign the informed consent due to the severity of the disease.

### Sociodemographic data

The general information about the patients included sex, age, height, weight, education, housing and financial situation, smoking status and alcohol consumption, physical diseases, symptoms, pain, medication and a history of suicidal ideation within the past month and the past 12 months.

### Questionnaires

For the assessment of the patient’s temperament the brief TEMPS-M auto-questionnaire was used [14], which differentiates five basic types of temperament: depressive, irritable, hyperthymic, anxious and cyclothymic [11, 12]. These affective dispositions are seen as biologically determined and time-stable traits of a person’s core personality [11, 12]. The brief TEMPS-M short version consists of 35 items, including 7 items for each type of temperament. It was developed and validated in in two independent German-speaking samples in Germany [14] and Austria [15]. Previous studies showed good internal consistency (alpha = 0.69–0.90) and sufficient validity (r = 0.49–0.72) for all subscales [14, 15].

The short 9‑item German version of the PHQ-9 adapted by Spitzer et al. in 1999 [16] was used to assess depressive and anxiety symptoms. For this study the analysis was based on the sum-score method. A PHQ-9 cut-off score ≥10 showed a pooled sensitivity of 85% and a pooled specificity of 89% for MDD [17]. In another meta-analysis, the pooled sensitivity for a cut-off score of 10 was 0.78 and the pooled specificity was 0.87, indicating acceptable diagnostic properties for MDD [18].

### Statistical methods

The statistical analysis was performed by IBM SPSS v. 24.0 (Microsoft, New Mexico, USA). The nonparametric Mann-Whitney U‑test was used for rank order between two groups. Associations between interval-scaled variables were tested by Spearman’s correlation coefficient.

## Results

### Sociodemographic data

Of the 55 participants 30 were female (54.5%) and 25 were male (45.5%), 46 (83.6%) participants were over 50 years and 9 (16.4%) were between 31 and 50 years old. The highest completed education was compulsory school in 4 participants (7.3%), vocational training in 22 participants (40%), secondary school in 15 participants (27.3%) and university degree in 10 participants (18.2%). Of the participants 4 (7.3%) did not indicate their educational status (Table 1) and 10 (18.2%) lived alone. None of the patients stated the financial situation as “not enough” and three participants (5.5%) did not indicate their financial situation (Table 1).

**Table 1:** Patient characteristics

*n*			55
*Sex*, *n (%)*		*Age (years)*, *n (%)*	
Male	25 (45.5)	31–50	9 (16.4)
Female	30 (54.5)	>50	46 (83.6)
*Education, n (%)*		*Smoker, n (%)*	
Compulsory	4 (7.3)	Yes	4 (7.3)
Vocational	22 (40)	Stopped smoking	30 (54.5)
Secondary	15 (27.3)	Never smoked	20 (36.4)
University	10 (18.2)	Missing	1 (1.8)
Missing	4 (7.3)		
*Housing situation*, *n (%)*		*Alcohol*, *n (%)*	
Living alone	10 (18.2)	Several times per week	3 (5.5)
Not living alone	39 (70.9)	Once a week	3 (5.5)
Missing	6 (10.9)	Less frequently	16 (29.1)
*Financial situation, n (%)*		Never	33 (60.0)
Very good	3 (5.5)		
Good	22 (40)		
Enough	27 (49.1)		
Missing	3 (5.5)		

As expected, higher education significantly correlated with a better financial situation (r = 0.482, *p* < 0.001). While the financial situation was significantly associated with depression scores measured by PHQ-9 (r = 0.348, *p* = 0.016), there were no associations with other somatic or mental symptoms including PHQ-9 anxiety (*p* < 0.05).

### Gender

Gender differences were not significant for self-reported psychiatric and somatic symptoms, PHQ-9, suicidal ideation and temperament (all *p* < 0.05), with the exception of the cyclothymic temperament, which was significantly more frequent among males (Z = −2.416, *p* = 0.016).

### Substance use patterns

In total only 7.3% (*n* = 4) were smokers, 30 participants (54.5%) had stopped smoking and 20 participants (36.4%) had never smoked. Of the participants 5.5% (*n* = 3) indicated drinking alcohol often but most patients reported drinking alcohol less frequently (*n* = 16, 29.1%) or never (*n* = 33, 60%; Table 1).

### Somatic diseases

Of the 55 patients 11 (20.8%) were diagnosed with pancreatic cancer, 9 (17.0%) suffered from lung cancer, 8 (15.1%) were diagnosed with breast cancer, 4 (7.6%) with renal cancer, 3 (5.7%) suffered from colorectal or ovarian cancer, 2 patients (3.8%) had either head and neck, gastric or esophageal cancer, biliary or prostate carcinoma. Furthermore, two patients suffered from cancer of unknown primary (CUP) and one patient each (1.9%) suffered from either malignant melanoma, multiple myeloma, hepatocellular cancer, sarcoma or mesothelioma.

### Self-reported somatic and mental symptoms

The assessment of psychiatric and somatic symptoms as shown in Table 2 suggests a high burden, with pain, anorexia, constipation and nausea/vomiting being the most prevalent self-reported symptoms in the sample. Interestingly, self-reported symptoms of depression were less commonly reported (21.8%) than unveiled by the structured assessment with PHQ-9.

**Table 2:** Distribution of self-reported somatic and psychiatric symptoms in patients (*N* = 55)

	*n*	%
Pain	37	67.3
Anorexia	32	58.2
Constipation	23	41.8
Nausea/vomiting	22	40.0
Edema	22	40.0
Anxiety	20	36.4
Dyspnea	20	36.4
Sleeplessness	18	32.7
Depression	12	21.8
Diarrhea	9	16.4
Confusion	4	7.3

### PHQ-9 and brief-TEMPS-M

Of the 55 patients, 53 PHQ-9 questionnaires on depressive symptoms were completed. Of these 26.4% (*n* = 14) reported mild symptoms of depression and 64.1% (*n* = 34) had a MDD. Only 8.5% (*n* = 4) showed no symptoms of depression. Out of 52 completed PHQ-9 questionnaires on anxiety symptoms, 34.6% (*n* = 18) showed symptoms of anxiety and 3.9% panic disorder. The patients also frequently reported suicidal ideations within the past month (23.6%, *n* = 13) and within the past 12-months (29.1%; *N* = 16).

As shown in Table 3 the depressive and cyclothymic temperaments showed significant associations with both depressive (both *p* < 0.001) symptoms and anxiety (*p* = 0.002; *p* = 0.036) as measured by PHQ-9. Furthermore, the anxious temperament was significantly associated with symptoms of depression (*p* = 0.027). All associations remained significant after controlling for the financial situation (all *p* < 0.05).

**Table 3:** Association of PHQ-9 score for depression and anxiety with affective temperament types

Correlation with temperament type		
	r	*p*-value
*Depression*		
Depressive	0.55	***p*** **<** **0.001**
Cyclothymic	0.52	***p*** **<** **0.001**
Hyperthymic	0.10	*p* = 0.501
Irritable	0.13	*p* = 0.366
Anxious	0.32	***p*** **=** **0.027**
*Anxiety*		
Depressive	0.43	***p*** **=** **0.002**
Cyclothymic	0.30	***p*** **=** **0.036**
Hyperthymic	0.16	*p* = 0.272
Irritable	0.02	*p* = 0.904
Anxious	0.16	*p* = 0.271

*Bold type* indicates depression or anxiety according to PHQ-9

**Fig. 1:**
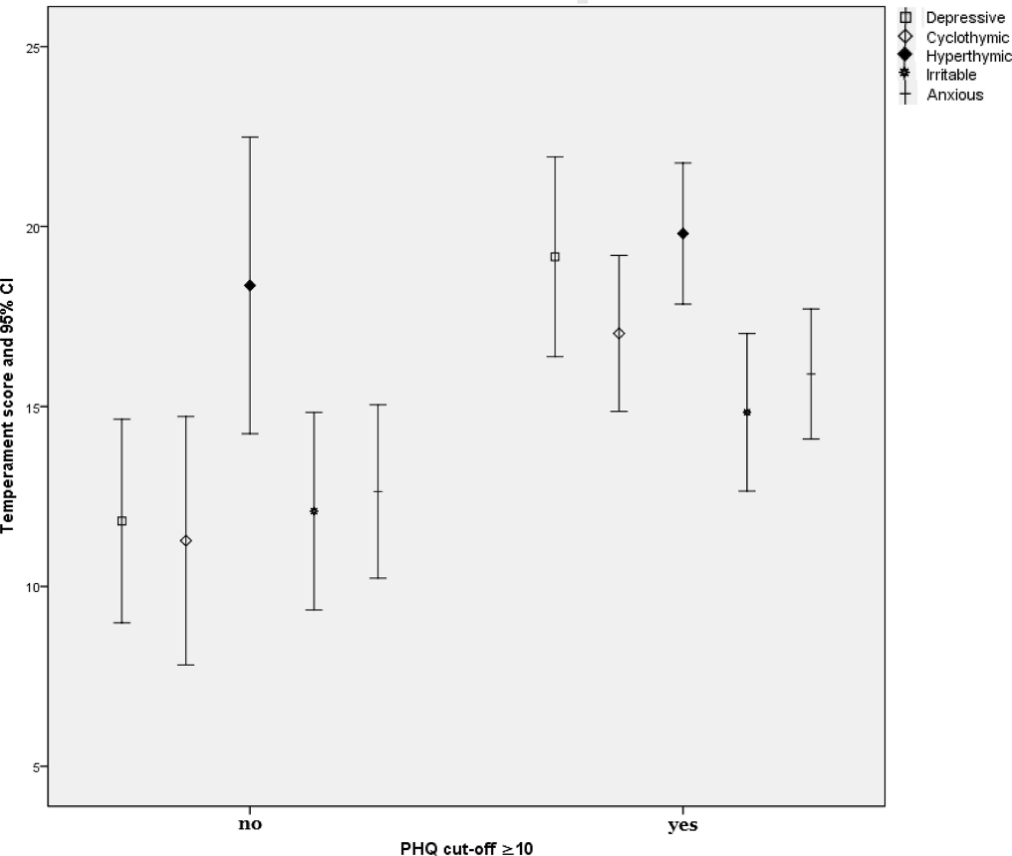
Differences in the temperament scores of the participants according to the PHQ(patient health questionnaire)-9 cut-off of ≥10

Fig. 1 shows the significant differences in temperament scores (depressive Z = −3.127, *p* = 0.002; cyclothymic Z = −2.810, *p* = 0.005; anxious Z = −2.013, *p* = 0.044) between depressive and non-depressive individuals as defined by the PHQ-9 cut-off ≥10. The irritable (Z = −1.191, *p* > 0.05) and hyperthymic temperaments (Z = −0.774, *p* > 0.05) showed no significant differences.

## Discussion

Psychiatric symptoms are common in palliative care patients, therefore, it is essential to evaluate the need for psychiatric and psychosocial support. Studies addressing the prevalence of depression in terminally ill patients have included cancer stage, histology, gender, age, performance status, comorbidity and marital status as confounding factors [1–4, 19]; however, the influence of temperament on depression and anxiety in PCU has not been assessed so far. In this study it was found that around 60% of patients suffered from MDD while 4% fulfilled the criteria for a panic disorder. A significant relationship was found between depressive, cyclothymic and anxious temperaments and depressive symptoms. Furthermore, a correlation between anxiety and the depressive as well as cyclothymic temperaments was observed.

Especially cancer patients who suffer from pain are frequently found to have increased rates of depression where psychiatric treatment is not initiated [20]. In a study with 90 patients with newly diagnosed non-small cell lung cancer and consecutively assessed in an oncology unit before starting with any treatment procedure, interviews within the first week of admission based on the Centre for Epidemiologic Studies Depression scale (CES-D) and the Temperament and Character Inventory (TCI), revealed that the temperament dimension, harm avoidance and physical pain were predictors for depression in patients with lung cancer [21]. According to other studies, specific dominant affective temperaments are predictive factors for the development and severity of depressive symptoms [22–25]. The results of this study imply that higher scores in the depressive and cyclothymic temperaments correlate with higher depression scores, hence supporting the hypothesis that affective temperament traits pose a risk factor for the development for depressive disorders, also in palliative care patients. In the current sample, MDD showed a high point prevalence of 64.1%, implicating a severe psychiatric burden of the sample and calling for appropriate psychiatric and psychosocial treatment.

### Limitations

With its cross-sectional design this study has some limitations. The rate of depression in this study is higher than in comparable studies. Lie et al. examined psychiatric symptoms in 969 palliative patients across Europe using the PHQ-9. They found a total rate of 45.3% for any depressive disorder [26]. This might be explained by the fact that self-report instruments generally show higher depression rates or also by the small sample size of consecutive patients within one clinic. Furthermore, patients were evaluated on arrival at the PCU. It is therefore possible that the time shortly after first admission to the palliative care unit might be a period of higher burden and may overlap with symptoms of adjustment disorder. Unfortunately the PHQ-9 cannot appropriately discriminate between depression and adjustment disorders, as many symptoms of both conditions overlap; however, from the perspective of clinical decision making, both adjustment disorder and MDD require appropriate psychiatric interventions. Another observation was the subjective overestimation that occurs when patients are answering questionnaires without an additional interview [27, 28]. To get an accurate psychiatric diagnosis a clinical interview should follow up the questionnaire [29]. Ryan et al. [30] reported that short screening instruments have a high sensitivity of 100% for distressed patients but relatively poor specificity (49–60%), and adding a short question to the screening about the need for help (e. g. “Is this something for which you would like help?”) could considerably increase the specificity in oncological settings. Further studies should also validate self-reported symptoms in a clinical face to face interview.

### Clinical implications

To the best of our knowledge, this is the first study which investigated the incidence of depression at a PCU with a focus on the individual temperament type. As a clinical implication depression is highly prevalent in terminally ill patients and due to its association with mortality and quality of life, screening for and appropriate treatment of depression within a multidisciplinary team is essential. This study could contribute to increase awareness for psychiatric vulnerabilities and it might be important to consider temperamental traits as risk factors in the etiology and therapy of depressive patients in palliative care. Further investigations to elaborate the concept of temperament in context to psychiatric disorders in palliative care are needed.

## References

[1] Carlson LE, Angen M, Cullum J (2004). High levels of untreated distress and fatigue in cancer patients. Br J Cancer.

[2] Masel EK, Berghoff AS, Mladen A (2016). Psyche at the end of life: psychiatric symptoms are prevalent in patients admitted to a palliative care unit. Palliat Support Care.

[3] Tu CH, Hsu MC, Chi SC (2014). Routine depression screening and diagnosing strategy for cancer inpatients. Psychooncology.

[4] Kerrihard T, Breitbart W, Dent R, Strout D (1999). Anxiety in patients with cancer and human immunodeficiency virus. Semin Clin Neuropsychiatry.

[5] Vignaroli E, Pace EA, Willey J (2006). The Edmonton Symptom Assessment System as a screening tool for depression and anxiety. J Palliat Med.

[6] Lo C, Zimmermann C, Rydall A (2010). Longitudinal study of depressive symptoms in patients with metastatic gastrointestinal and lung cancer. J Clin Oncol.

[7] Satin JR (2010). Review: depression is associated with increased cancer mortality. Evid Based Ment Health.

[8] Blöink R (2005). Factorial structure and internal consistency of the German TEMPS-A scale: validation against the NEO-FFI questionnaire. J Affect Disord.

[9] Solmi M, Zaninotto L, Toffanin T (2016). A comparative meta-analysis of TEMPS scores across mood disorder patients, their first-degree relatives, healthy controls, and other psychiatric disorders. J Affect Disord.

[10] Kampman O, Viikki M, Leinonen E (2017). Anxiety disorders and temperament—an update review. Curr Psychiatry Rep.

[11] Akiskal HS (2002). Temperament und affektive Störungen. Die TEMPS-A-Skala als Konvergenz europäischer und US-amerikanischer Konzepte. Nervenarzt.

[12] Akiskal HS (2005). TEMPS-A: validation of a short version of a self-rated instrument designed to measure variations in temperament. J Affect Disord.

[13] Kesebir S, Gündoğar D, Küçüksubaşı Y (2013). The relation between affective temperament and resilience in depression: a controlled study. J Affect Disord.

[14] Erfurth A (2005). Studies on a German (Münster) version of the temperament auto-questionnaire TEMPS-A: construction and validation of the briefTEMPS-M. J Affect Disord.

[15] Naderer A, Keller F, Plener P (2015). The brief TEMPS-M temperament questionnaire: a psychometric evaluation in an Austrian sample. J Affect Disord.

[16] Spitzer R, Kroenke K, Williams J (1999). Validation and utility of a self-report version of PRIME-MD. The PHQ primary care study. J Am Med Assoc.

[17] Manea L, Gilbody S, McMillan DA (2015). Diagnostic meta-analysis of the Patient Health Questionnaire-9 (PHQ-9) algorithm scoring method as a screen for depression. Gen Hosp Psychiatry.

[18] Moriarty AS, Gilbody S, McMillan D (2015). Screening and case finding for major depressive disorder using the Patient Health Questionnaire (PHQ-9): a meta-analysis. Gen Hosp Psychiatry.

[19] Nekolaichuk CL, Bruera E, Spachynski K (1999). A comparison of patient and proxy symptom assessments in advanced cancer patients. Palliat Med.

[20] Tomita T (2014). An investigation of temperament and character inventory items for predicting the response to paroxetine treatment in patients with major depressive disorder. J Affect Disord.

[21] Aukst M, Kukulj S, Šantić Ž (2013). Predicting depression with temperament and character in lung cancer patients. Eur. J. Cancer Care (Engl.).

[22] Grucza RA, Przybeck TR, Spitznagel EL (2003). Personality and depressive symptoms: a multi-dimensional analysis. J. Affect. Disord..

[23] Hansenne M, Pitchot W, Pinto E (1999). Temperament and character inventory (TCI) and depression. J Psychiatr Res.

[24] Boz C, Velioglu S, Ozmenoglu M (2004). Temperament and character profiles of patients with tension-type headache and migraine. Psychiatry Clin Neurosci.

[25] Celikel FC, Kose S, Cumurcu B (2009). Cloningers temperament and charater dimensions of personality in patients with major depressive disorder. Compr Psychiatry.

[26] Lie HC, Hjermstad MJ, Fayers P (2015). Depression in advanced cancer—assessment challenges and associations with disease load. J Affect Disord.

[27] Rabkin J, McElhiney M, Moran P (2009). Depression, distress and positive mood in late-stage cancer: a longitudinal study. Psychooncology.

[28] Mehnert A, Brähler E, Faller H (2014). Four-week prevalence of mental disorders in patients with cancer across major tumor entities. J Clin Oncol.

[29] Chan C, Ahmad W, Yusof M (2015). Effects of depression and anxiety on mortality in a mixed cancer group: a longitudinal study approach using standardised diagnostic interviews. Psychooncology.

[30] Ryan DA, Gallagher P, Wright S, Cassidy EM (2012). Sensitivity and specificity of the Distress Thermometer and a two-item depression screen (Patient Health Questionnaire-2) with a ‘help’ question for psychological distress and psychiatric morbidity in patients with advanced cancer. Psychooncology.

